# Circulating microRNA-92a and microRNA-21 as novel minimally invasive biomarkers for primary breast cancer

**DOI:** 10.1007/s00432-012-1315-y

**Published:** 2012-09-30

**Authors:** Haiyan Si, Xiaoming Sun, Yingjian Chen, Yuan Cao, Shimin Chen, Huanchun Wang, Chengjin Hu

**Affiliations:** Department of Laboratory Medicine, Jinan General Hospital of PLA, Jinan, 250031 Shandong People’s Republic of China

**Keywords:** Breast cancer, microRNA, miR-92a, miR-21, KLK5, qRT-PCR

## Abstract

**Purpose:**

MicroRNAs (miRNAs) play an essential role in breast malignant tumor development and progression. The development of clinically validated biomarkers for primary breast cancer (BC) has remained an insurmountable task despite other advances in the field of cancer molecular biology. The objective of this study is to investigate the differential expression of miRNAs and the potential of circulating microRNAs as novel primary breast cancer biomarkers.

**Methods:**

Our analyses were performed on 48 tissue and 100 serum samples of patients with primary BC and a set of 20 control samples of healthy women, respectively. The relative expression of ten candidate miRNAs (miR-106b, miR-125b, miR-17, miR-185, miR-21, miR-558, miR-625, miR-665, miR-92a, and miR-93) from the results of four bioinformatics approaches and literature curation was measured by real-time quantitative reverse transcription PCR (qRT-PCR).

**Results:**

The level of miR-92a was significantly lower, while miR-21 was higher, as previous reports, in tissue and serum samples of BC than that of healthy controls (*p* < 0.001). Logistic regression and receiver operating characteristic curve analyses revealed the significant and independent value (*p* < 0.001) of the miR-92a and miR-21 expression quantification in serums. Moreover, the comparison with the clinicopathologic data of the BC patients showed that decreased levels of miR-92a and increased levels of miR-21 were associated with tumor size and a positive lymph node status (*p* < 0.001).

**Conclusions:**

These findings suggest that many miRNAs expressions are altered in BC, whose expression profiling may provide a useful clue for the pathophysiological research. Circulating miR-92a has potential use as novel breast cancer biomarker, which is comparable to miR-21.

## Introduction

Development of breast malignant tumor is complex multistep process associated with numerous genetic alterations. MicroRNAs (miRNAs) are small, non-coding RNA molecules, which modulate expression of target genes and play essential roles in biological and pathological processes of diseases (Esquela-Kerscher and Slack 2006). miRNAs regulate posttranscriptional gene expression depending upon the complementarities of their sequences with target mRNAs (Bartel 2004). Kallikrein-related peptidase 5 (KLK5) is a secreted trypsin-like serine protease, encoded by the human kallikrein gene 5 (*KLK5*) of the kallikrein family, under the transcriptional control of estrogens and progestins (Yousef and Diamandis 1999). It has been reported that this gene is mainly expressed in breast and brain (Yousef and Diamandis 1999; Brattsand and Egelrud 1999). The prognostic value of *KLK5* expression had already been demonstrated for BC (Yousef et al. 2002, 2004; Avgeris et al. 2011). In our previous work, we have confirmed that a statistically significant down-regulation of the *KLK5* expression levels was observed in the malignant breast specimens compared with the benign ones.

The diagnostic and prognostic value of *KLK5* has already been demonstrated in BC patients; however, miRNAs which may regulate *KLK5* expression have not been identified, and whether they can serve as potential biomarkers of BC is still unclear. In our previous work, hundreds of miRNAs are predicted by four bioinformatics approaches including TargetScan Human 5.2, MicroCosm Targets Version 5, ExprTarget, and MIRANDA (Lewis et al. 2005; Griffiths-Jones et al. 2008; Gamazon et al. 2010; Lagos-Quintana et al. 2001). With the results above and literature curation, ten putative miRNAs (miR-106b, miR-125b, miR-17, miR-185, miR-21, miR-558, miR-625, miR-665, miR-92a, and miR-93) are selected to validate. Numerous publications have been reported that circulating miRNAs may serve as stable blood-based biomarkers in carcinomas (Schwarzenbach et al. 2011; Mitchell et al. 2008; Heneghan et al. 2010). Therefore, we detect the expression levels of the ten miRNAs in the tissue and serum samples of BC patients and investigate the potential of circulating microRNAs as novel primary breast cancer biomarkers.

## Methods

### Study subjects

Tumor tissues, paired normal adjacent tissues (NATs), and matching serum samples were collected from 48 cases of patients with newly diagnosed breast carcinomas. Other serum samples came from 52 cases of BC patients and 20 cases of healthy controls (HC). All the samples were recruited at Jinan General Hospital of PLA from January 2009 to December 2011. The clinical stage was classified according to the American Joint Committee on Cancer (AJCC) tumor-lymph node-metastasis (TNM) classification system. This work was performed according to the guidelines of Jinan General Hospital of PLA, which abides by the Helsinki Declaration on ethical principle for medical research involving human subjects. All subjects gave informed consent to this work.

### Total RNA isolation

The miRNeasy Mini Kit (Qiagen, USA) and mirVana PARIS Kit (Ambion, USA) were used to isolate the total RNA from the tissue and serum samples according to the manufacture's protocols. RNA quality and quantity were measured by Lambda Bio UV/VIS Spectrophotometer (PERKIN ELMER, USA). The RNA samples were immediately stored at −80 °C or converted into cDNA immediately.

### miRNA quantification by qRT-PCR

SYBR green qRT-PCR assay was used for miRNA quantification. In brief, 40–500 ng of RNA containing miRNA was polyadenylated by poly(A) polymerase and reverse transcribed to cDNA by One Step PrimeScript^®^ miRNA cDNA Synthesis Kit (TaKaRa, China) according to the manufacturer’s instructions. Twenty-microliter reverse transcriptase reactions contained 100 ng total RNA derived from tissue or 40 ng total RNA form serum, 10 μl 2× miRNA Reaction Buffer Mix (including dNTP Mixture, Mg^2+^ and Universal Adaptor Primer), 2 μl 0.1 % BSA, 2 μl miRNA PrimeScript^®^ RT Enzyme Mix, and RNase Free dH_2_O up to 20 μl. The mixture was incubated at 50 °C for 60 min and 85 °C for 5 s with XP CYCLER (BIOER, China).

Real-time quantitative PCR was performed by Roche LightCycler480 II (Roche, Switzerland) with SYBR^®^ Premix Ex Taq™ II kit (TaKaRa, China). The miRNA-specific forward primers were designed by Primer Premier 5.0, and the sequences are shown in Table 1. The reverse primers were provided by the SYBR^®^ Premix Ex Taq™ II kit (Uni-miR qPCR Primer). The reaction was performed at 95 °C for 30 s, followed by 40 cycles at 95 °C for 5 s and 60 °C for 20 s, and then ramped from 65 to 95 °C to obtain the melting curve. RNU6 was assessed as the reference control for tissue studies, and miR-16 was for serum studies. The obtained data of miRNA expression levels are calculated and evaluated by the ΔCT method as follows: ΔCT = Ct(miRNA of interest) − Ct(reference of control). The relative expression of the target miRNA was calculated by the method: (1 + *E*)^(−ΔCT)^, *E*: amplification efficiency. The Ct value was the threshold cycle to detect fluorescence. Each sample was run in duplicates for analysis.

**Table 1:** miRNA-specific forward primer sequences

Gene name	Primer sequence
miR-106b	5′-CGTAAAGTGCTGACAGTGCAGAT-3′
miR-125b	5′-GTCCCTGAGACCCTAACTTGTGA-3′
miR-17	5′-CAAAGTGCTTACAGTGCAGGTAG-3′
miR-185	5′-TGGAGAGAAAGGCAGTTCCTGA-3′
miR-21	5′-GGCGTAGCTTATCAGACTGATGTTG-3′
miR-558	5′-CGCTGAGCTGCTGTACCAAAAT-3′
miR-625	5′-CCAGGGGGAAAGTTCTATAGTCC-3′
miR-665	5′-ACCAGGAGGCTGAGGCCCCT-3′
miR-92a	5′-TATTGCACTTGTCCCGGC-3′
miR-93	5′-CAAAGTGCTGTTCGTGCAGG-3′
RNU6	5′-TCGTGAAGCGTTCCATATTTTT-3′
miR-16	5′-CAGCACGTAAATATTGGCG-3′

### Statistical analysis

The statistical analysis was performed by the SPSS software package, version 17.0 (SPSS Inc. USA). Mann–Whitney* U* test, Wilcoxon test, ANOVA, LSD analysis, Spearman-Rho test, univariate logistic regression analysis, and receiver operating characteristic (ROC) curves were used in this work. A *p-*value <0.05 was considered as statistically significant. All *p* values were two-sided.

## Results

### miRNAs expression in the tissue samples of BC and NATs

**Fig. 1:**
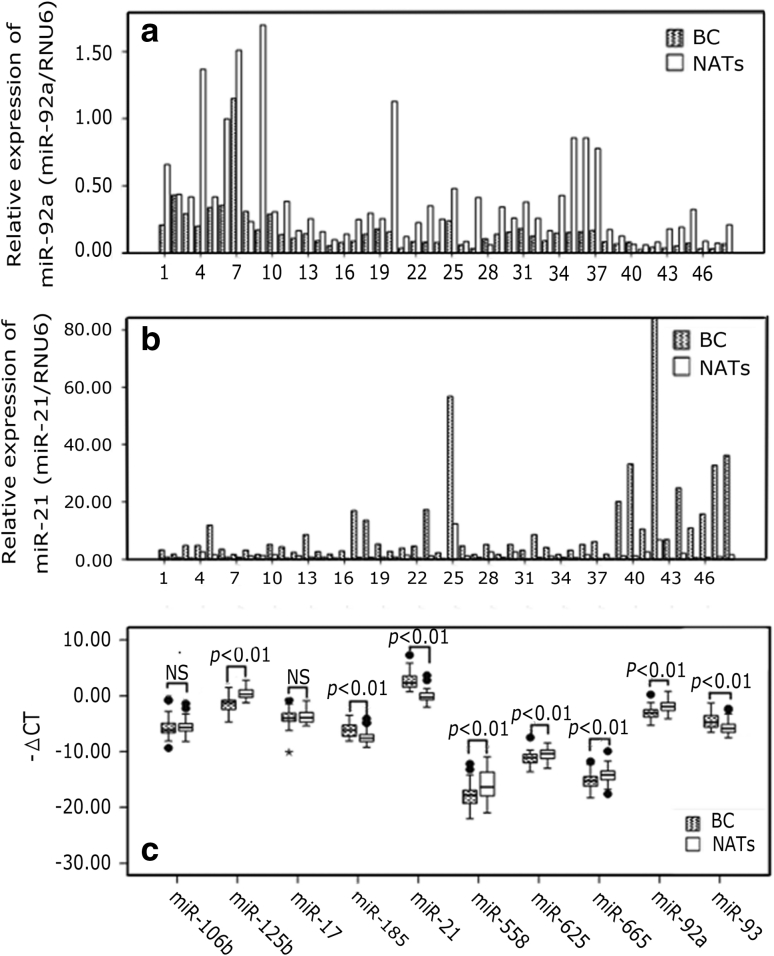
Expression of miR-92a (**a**) and miR-21(**b**) in BC and matched NATs No. 1–48, (*n* = 48). AJCC I, No. 1–10 (*n* = 10); AJCC II, No. 11–37 (*n* = 27); AJCC III, No. 38–48 (*n* = 11). Relative expression levels of miRNAs were determined by calculating the ratio between the miRNA and RNU6. **c** Relative expression levels of the ten miRNAs in BC and matched NATs are shown as* box plots*. The *horizontal lines* represent the *median*, the *bottom* and *top* of the *boxes* represent the 25th and 75th percentiles, respectively, and the *vertical bars* represent the range of data. “*filled circle*” over the 1.5 folds of the interquartile range; “*asterisk*”: over the threefold of the interquartile range; −ΔCT = Ct(reference RNU6) − Ct(miRNA of interest); NS: *p* > 0.05; The significant *p* values of the statistical evaluations are indicated *above* the* plots*

In the present study, we examined the expression of 10 miRNAs in BC tissues and the paired NATs. With Wilcoxon test of two dependent variables, miR-185, miR-21, and miR-93 were significantly overexpressed in the tumor specimens compared with those in NATs, whereas miR-125b, miR-558, miR-625, miR-665, and miR-92a were significantly down-expressed in BC (*p* < 0.01). The expression of miR-106b and miR-17 in BC tissue samples were not significantly different from the NATs (*p* > 0.05) (Fig. 1).

### miRNAs expression in the serum samples of BC and HC

**Fig. 2:**
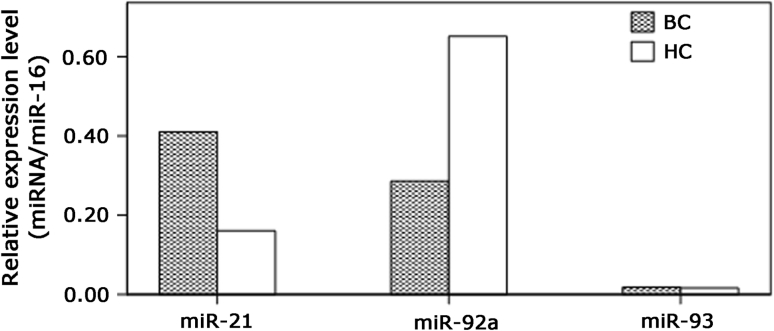
Expression of miR-21, miR-92a, and miR-93 in BC and HC. Total RNA derived from the samples of BC (*n* = 100), and HC (*n* = 20) was subjected to qRT-PCR. Relative expression levels were determined by calculating the ratio between the target and the control miRNA

Preliminary experiments were employed to evaluate the Ct values of the ten miRNAs in serum samples. Seven miRNAs including miR-106b, miR-125b, miR-17, miR-185, miR-558, miR-625, and miR-665 were removed for low expression with the Ct value about 35. The rest of the miRNAs including miR-21, miR-92a, and miR-93 were examined in the serum samples of BC and HC. Consistent with the aberrant expression in BC tissue samples, miR-21 and miR-92a showed similar pattern of expression change in serum samples, while miR-93 had no significant difference between the serum samples of BC and HC. The results are displayed in Fig. 2.

### The relationship between miR-92a and miR-21 expressions and clinical histopathological features

**Fig. 3:**
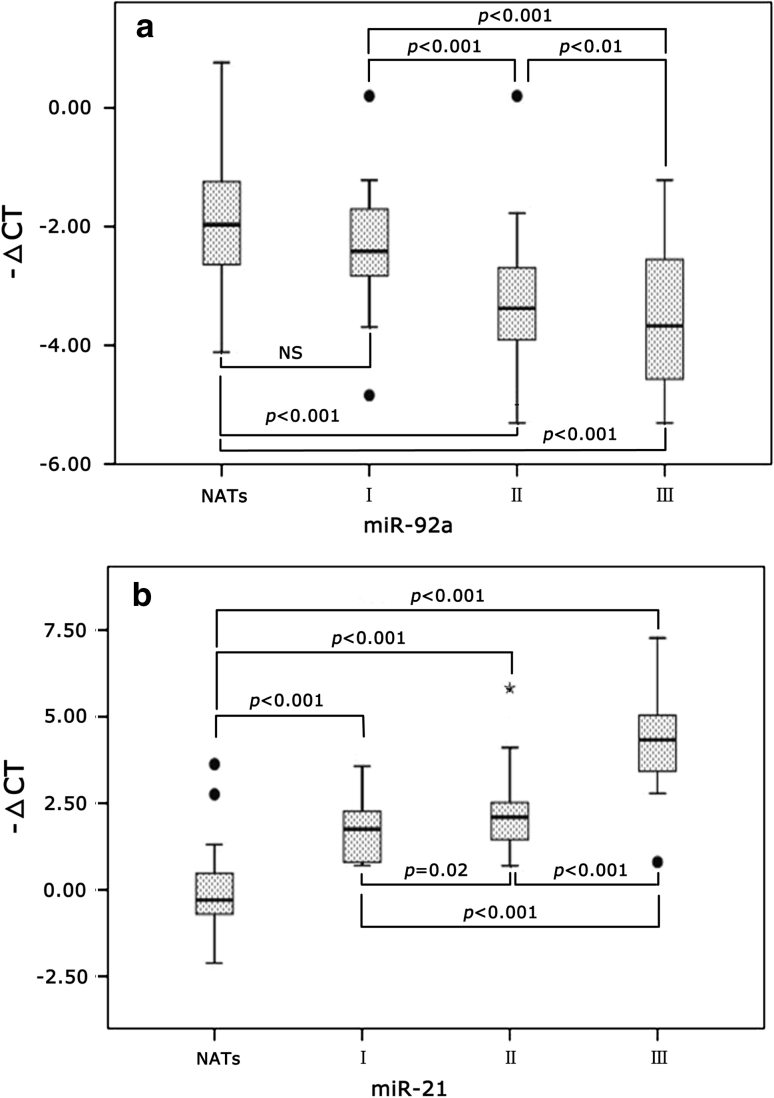
Relative expressions of miR-92a (**a**) and miR-21 (**b**) in NATs and BC AJCC stages are shown as *box plots*. The subjects were divided into four groups based on the AJCC tumor-lymph node-metastasis classification system, representing I (*n* = 10), II (*n* = 27), III (*n* = 11) and NATs (*n* = 48). −ΔCT = Ct(reference RNU6) − Ct(miRNA of interest); NS: *p* > 0.05. The significant *p* values of the statistical evaluations are indicated *above* the* plots*

The differences in expression of miR-21 and miR-92a in NATs, BC tissues of AJCC stage I, II, III were analyzed by ANOVA and LSD analysis, and the results are shown in Fig. 3. The relationship between miR-92a and miR-21 expressions and clinical histopathological features was analyzed by Mann–Whitney* U* test, and the results are shown in Table 2. There were no significant differences between the expressions of the remained miRNAs and clinical histopathological features. The expressions of the ten miRNAs have no significant difference among the patients’ ages (*p* > 0.05).

**Table 2:** The relationship between the relative expression levels of miR-92a and miR-21 and the clinical histopathological features

Variable	No	Relative expression level of miR-92a (95 % CI)	*p*	Relative expression level of miR-21 (95 % CI)	*p*
*Tissues*					
BC	48	0.11–0.21	<0.01	5.30–19.08	<0.01
NATs	48	0.28–0.51		0.79–1.90	
*Serums*					
BC	100	0.20–0.37	<0.01	0.32–0.51	<0.01
HC	20	0.50–0.80		0.13–0.19	
*Tumor size*					
<2 cm	10	0.10–0.54	<0.01	1.12–11.40	<0.01
≥2 cm	38	0.09–0.14		5.11–22.39	
*Lymph node metastasis*					
Negative	28	0.13–0.29	<0.01	1.88–9.81	<0.01
Positive	20	0.06–0.10		5.53–36.60	
*Estrogen receptor*					
Negative	17	0.08–0.15	>0.05	4.21–20.00	>0.05
Positive	31	0.10–0.26		2.14–22.32	
*Progesterone receptor*					
Negative	8	0.04–0.17	>0.05	3.17–36.93	>0.05
Positive	40	0.11–0.23		2.85–18.38	
*CerbB*-*2*					
Negative	39	0.10–0.18	>0.05	3.18–27.22	>0.05
Positive	9	0.07–0.28		3.87–12.76	

### The correlation of miR-21 and miR-92a expression pattern between BC tissues and paired serums

Spearman-Rho test was carried out to compare the relative expression of miR-21 and miR-92a in 48 cases of BC tissues and paired serums. The results showed a significant correlation of miR-21 expression profiles in the tissues with those in the serums, with *r* = 0.61 and miR-92a with *r* = 0.51 (*p* < 0.001).

### Estimation of the predictive value of miR-92a and miR-21 expression regarding the presence of BC

**Fig. 4:**
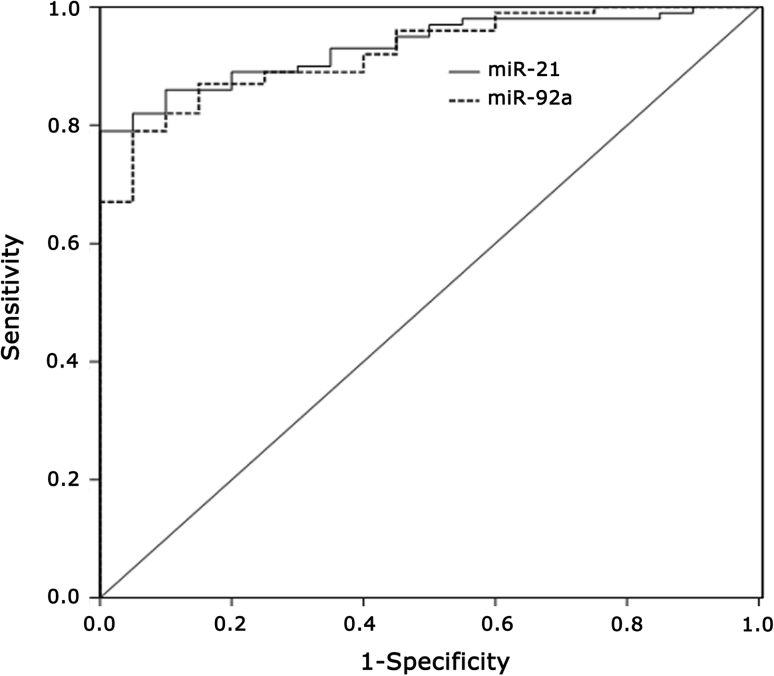
miR-92a and miR-21 were plotted to discriminate HC and BC patients. miR-92a and miR-21 yield an area under the curve (AUC) value of 0.923 and 0.933, respectively

To investigate the predictive value of miR-92a and miR-21 in BC, we measured their expression levels in serum samples of 100 BC and 20 HC. The results by univariate logistic regression analysis showed the significant negative correlation between the miR-92a expression levels and the risk of a patient to suffer for BC (*p* < 0.001). Moreover, patients with low miR-92a expression levels were at a higher risk of breast malignancy as compared to those with high miR-92a expression profiles (HR 0.07); meanwhile, patients with high miR-21 expression levels were at a higher risk of breast malignancy as compared to those with low miR-21 expression profiles (HR 10.07). ROC curve analyses also showed that both miRNAs could differentiate BC from HC with an AUC of 0.923 for miR-92a (95 % CI 0.873–0.973) and 0.933 for miR-21 (95 % CI 0.889–0.977), respectively (Fig. 4).

## Discussion

miRNAs are small regulatory RNAs involved in various physiological and pathophysiological processes (Osaki et al. 2008). Recently, miRNAs show great potential as diagnostic and prognostic biomarkers for BC (Lowery et al. 2008; Iorio et al. 2005; Blenkiron et al. 2007; Heneghan et al. 2011). In this work, the levels of ten candidate miRNAs (miR-106b, miR-125b, miR-17, miR-185, miR-21, miR-558, miR-625, miR-665, miR-92a, and miR-93) are quantified by qRT-PCR in tissue and serum samples of patients with BC as well as HC.

The previous study suggests that miRNAs are stable in serum by time-course and freeze–thaw cycle analyses and can escape from RNAse degradation (Schwarzenbach et al. 2011; Mitchell et al. 2008). miRNAs were for the first time described and detected in the serum of patients with diffuse B-cell lymphoma in the year 2008 (Lawrie et al. 2008). Roth et al. (2010) had confirmed that tumor-associated circulating miRNAs were elevated in the blood of BC patients and associated with tumor progression. The relative concentrations of miR-155 in serum significantly discriminated primary BC patients from healthy women, whereas miR-10b, miR-34a, and miR-155 discriminated metastatic disease patients from HC. Asaga et al. (2011) had proved that circulating miR-21 had diagnostic and prognostic potential in BC. miR-214 was certified as a diagnostic potential indicator in BC for malignant disease and metastatic (Schwarzenbach et al. 2012). Because the serum samples could be easily obtained at different time points during the course of the disease and the circulating miRNAs could be quantified by qRT-PCR (Kroh et al. 2010), circulating miRNAs as novel minimally invasive indicators have important applications in cancer detection. Serum miRNA is currently limited, due to the fact that large RNA molecules are detected to be degraded or relatively high Ct values in serum samples. In our work, miR-106b, miR-125b, miR-17, miR-185, miR-558, miR-625, and miR-665 have low expression levels with the Ct values about 35 in serums. Just miR-21, miR-92a, and miR-93 can be quantified by the qRT-PCR in serum samples.

miR-21 is one of the most significantly up-regulated miRNAs in human BC, and its expression had been associated with tumor progression and poor prognosis. In our research, miR-21 is significantly up-regulated in the BC tissues and serums, and the results indicate a trend for the association of high miR-21 expression with poor patient survival. Our results of the miR-21 are consistent with the prior studies (Asaga et al. 2011; Qian et al. 2009). Studies have demonstrated that miR-21 functions as an oncogene by targeting tumor suppressor genes including tropomyosin 1 (TPM1), programmed cell death 4 (PDCD4), and phosphatase and tensin homolog (PTEN), leading to cell proliferation and inhibition of apoptosis and regulating cancer invasion and metastasis in breast cancer (Zhu et al. 2007; Frankel et al. 2008; Yan et al. 2008).

miR-92a is one of the seven mature miRNAs (miR-17, miR-18a, miR-19a, miR-20a, miR-19b, and miR-92a) encoded by the miRNA-17–92 cluster. The miR-17–92 cluster raises first interests after several studies linked the expression of the cluster to cancer pathogenesis (Mendell 2008). It had been reported that miR-92a promoted lymph node-metastasis of human esophageal squamous cell carcinoma (ESCC) via E-Cadherin (Chen et al. 2011). Ohyashiki et al. (2011) reported that the expression level of miR-92a was down-regulated in non-Hodgkin’s lymphoma (NHL). Plasma miR-92a value in NHL was extremely lower compared with that in healthy subjects, irrespective of lymphoma subtype. Tsuchida et al. (2011) determined that miR-92a was transcribed at a higher level in both adenomas and carcinoma. miR-92a directly targeted the anti-apoptotic molecule BCL-2-interacting mediator of cell death in colon cancer, and they indicated that miR-92a played a pivotal role in the development of colorectal carcinoma. Our, hitherto, investigations demonstrate the cancer-specific decrease in levels of miR-92a. In addition, the decreased level of miR-92a is also associated with positive lymph node status, indicating that miR-92a might be involved in cancer progression. Thus, our findings suggest that quantification of miR-92a might be suitable for detecting BC and lymph node metastases. So far, no investigation of miR-92a has been carried out in blood and tissue samples from BC patients.

In order to determine the correlation of miRNA expression in tissue and the matched serum samples, our results show a significant correlation of miR-21 and miR-92a expression profiles in the tissues with those in the serums (Spearman-Rho test *r* = 0.61 and 0.51, *p* < 0.01), which suggests that miR-21 and miR-92a isolated from serums could reflect most of the characteristic expression patterns of their tissue counterparts and further show promise for miRNAs as blood-based biomarkers for detecting and screening breast tumors. The univariate logistic regression model discloses a statistically significant elevated risk of the patients with reduced miR-92a and increased miR-21 expression to suffer from BC (*p* < 0.001). As illustrated by the ROC curves with AUC values of 0.923 for miR-92a and 0.933 for miR-21, respectively, it suggests that miR-92a and miR-21 expression quantification may be used to discriminate the BC patients from the HC.

At present, miRNA have become the rising stars in cancer genetics. miRNAs are excellent candidates for novel molecular targeting treatments because of their ability to regulate multiple genes in molecular pathways. Chemotherapy is an important component in the treatment paradigm for cancers. However, the resistance of cancer cells to chemotherapeutic agents frequently results in the subsequent recurrence and metastasis. Recently, a new data suggest that the expression level of miR-21 in tumor tissue and plasma might be used as a biomarker to predict adjuvant platinum-based chemotherapy response and disease-free survival in patients with non-small-cell lung cancer. Thus, it may serve as a novel therapeutic target to modulate platinum-based chemotherapy (Gao et al. 2012). Wang et al. (2012) had demonstrated that circulating miR-125b expression was associated with chemotherapeutic resistance of breast cancer. This finding has important implications in the development of targeted therapeutics for overcoming chemotherapeutic resistance in novel anticancer strategies. Trastuzumab resistance emerges to be a major issue in anti-HER2 therapy for breast cancers. Gong et al. (2011) had demonstrated that miR-21 overexpression contributes to trastuzumab resistance in HER2+ breast cancers. PTEN was identified as a miR-21 target. Administering miR-21 antisense oligonucleotides restored trastuzumab sensitivity in the resistant breast cancer xenografts by inducing PTEN expression, whereas injection of miR-21 mimics conferred trastuzumab resistance in the sensitive breast tumors via PTEN silence. Resistance to docetaxel also occurred in 50 % of breast cancer patients.

In this work, miRNAs expressions are altered in BC, whose expression profiling may provide a useful clue for the pathophysiological research. The expression level of miR-92a has potential predictive value as novel breast cancer biomarker in serum samples of BC and correlates with tumor size and lymph node metastases. The study of miRNAs may lead to finding their potential for improving diagnosis, prognosis, and their impact on future therapeutic strategies.
